# Triterpenoids of Three Apple Cultivars—Biosynthesis, Antioxidative and Anti-Inflammatory Properties, and Fate during Processing

**DOI:** 10.3390/molecules28062584

**Published:** 2023-03-13

**Authors:** Łukasz Woźniak, Anna Szakiel, Agnieszka Głowacka, Elżbieta Rozpara, Krystian Marszałek, Sylwia Skąpska

**Affiliations:** 1 Department of Food Safety and Chemical Analysis, Institute of Agricultural and Food Biotechnology—State Research Institute, 36 Rakowiecka Street, 02532 Warsaw, Poland; 2 Department of Plant Biochemistry, Faculty of Biology, University of Warsaw, 1 Miecznikowa Street, 02096 Warsaw, Poland; 3 Department of Pomology, Gene Resources and Nursery, The National Institute of Horticulture Research, 1/3 3 Maja Street, 96100 Skierniewice, Poland; 4 Department of Fruit and Vegetable Product Technology, Institute of Agricultural and Food Biotechnology—State Research Institute, 36 Rakowiecka Street, 02532 Warsaw, Poland

**Keywords:** antioxidants, cuticular waxes, cyclooxygenases, GC-MS, Golden Delicious, Ligol, *Malus domestica*, phytosterols, Redkroft, triterpenes, ursolic acid

## Abstract

Triterpenoids are a group of secondary plant metabolites, with a remarkable pharmacological potential, occurring in the cuticular waxes of the aerial parts of plants. The aim of this study was to analyze triterpenoid variability in the fruits and leaves of three apple cultivars during the growing season and gain new insights into their health-promoting properties and fate during juice and purée production. The identification and quantification of the compounds of interest were conducted using gas chromatography coupled with mass spectrometry. The waxes of both matrices contained similar analytes; however, their quantitative patterns varied: triterpenic acids prevailed in the fruits, while higher contents of steroids and esterified forms were observed in the leaves. The total triterpenoid content per unit area was stable during the growing season; the percentage of esters increased in the later phases of growth. Antioxidative and anti-inflammatory properties were evaluated with a series of in vitro assays. Triterpenoids were found to be the main anti-inflammatory compounds in the apples, while their impact on antioxidant capacity was minor. The apples were processed on a lab scale to obtain juices and purées. The apple purée and cloudy juice contained only some of the triterpenoids present in the raw fruit, while the clear juices were virtually free of those lipophilic compounds.

## 1. Introduction

The cuticle is a complex hydrophobic layer covering the non-woody aerial parts of plants, which has been developed through evolution to allow plants to survive in a terrestrial environment and endure its challenges. Consequently, the cuticle’s main function is to act as a barrier to transpirational loss of water, although it is also responsible for protecting the plant against pests and pathogens, screening UV-B radiation, and defining organ boundaries during development [1]. The typical composition of cuticles includes a macromolecular scaffold of linked cutin and a variety of lipids that are collectively termed waxes. The chemical composition of the waxes shows great variability between species, but also among the organs of the same plant, development stages, and environmental conditions [2,3,4]. The primary constituents of waxes are very long chain fatty acids (typically C20-C34) and their derivatives, which are responsible for the mechanical and waterproof properties of cuticles. In addition to these, cuticles often contain secondary metabolites, the most important of which are triterpenoids [1,5].

The term “triterpenoids” is used to describe the group of plant metabolites synthetized from a common intermediate, 2,3-oxidosqualene. Its cyclization and rearrangement lead to the formation of two groups of metabolites: sterols (containing a tetracyclic scaffold and a side chain) and triterpenes (containing a pentacyclic scaffold), while further enzymatic modifications result in the occurrence of more than 20,000 different structures found in nature [6]. Triterpenes from the oleane, ursane, and lupane families are the most common constituents of cuticular waxes [7]. They have important ecological and agronomical significance connected with resistance against pests and pathogens; additionally, their impact on consumer health is considered very promising [3,6]. Ursolic acid, which is widely recognized as being the most abundant of the triterpenoids present in apples, exhibits a wide array of pharmacological activities. The features of ursolic acid include anti-cancer potential, antioxidative and anti-inflammatory properties, protection of internal organs against chemically induced damage, and anti-microbial potential directly connected with the acid’s role in plants’ defense system [8]. Plant sterols (phytosterols) can also be found in plant cuticles, although, unlike triterpenes, they are ubiquitous in plant cells since they are responsible for the stabilization of cellular membranes. Dietary phytosterols are important agents in the prevention and treatment of hypercholesterolemia, and in a wider context, they have a significant impact on the absorption of fat-soluble diet components [9]. Plant peels can also contain other groups of secondary metabolites, such as anthocyanins and lycopene, which are responsible for coloration, although their presence was not investigated in this study.

Apples are among the most widely produced and consumed fruits worldwide, with approximately 80 million tons harvested each year [10]. They are the most important fruit considering the total revenue of their sale, while the cultivation, trade, and processing of apples provide maintenance to thousands of people around the world [11]. In addition to their nutritional value and economic significance, apples are also a rich source of secondary metabolites with bioactive potential, which are claimed to have a beneficial impact on health [12,13]. Thus far, the majority of the research has been focused on apple polyphenols, which include, inter alia, chlorogenic acid, catechin, epicatechin, phloridzin, and procyanidins [14]. The polyphenol levels are considered the main quality parameter in studies evaluating apples’ nutraceutical composition and improvement possibilities [15]. In recent years, an increasing number of publications have reported the content and properties of the lipophilic compounds present in the cuticular waxes of apples. The majority of the data in the literature [16,17,18,19,20] show that the typical triterpenoid pattern of apple peel includes ursolic acid as the dominant compound, followed by oleanolic acid; however, many contradictory results have been published over the years, which justifies further investigation. Additionally, the formation of triterpenoid esters with various compound groups (fatty acids, carbohydrates, phenolic acids) has also been reported [21,22,23,24], but these compounds are often overlooked during analysis.

The aim of this work was to analyze the triterpenoid content in cuticular waxes encompassing the fruits and leaves of apples and its changes during the growing season. The experiments included both free and esterified forms of triterpenic acids, neutral triterpenes, and sterols in three apple cultivars from the same origin. This paper broadens the knowledge on the presence of triterpenoids in apples and, according to the authors’ best knowledge, is the first work that investigates the content of these compounds in apple leaves. Furthermore, the focus was placed on the biological activities of the triterpenoids. Their antioxidative and anti-inflammatory properties were analyzed in vitro, including their stability during processing.

## 2. Results

### 2.1. Identification of Triterpenoids

**Figure 1:**
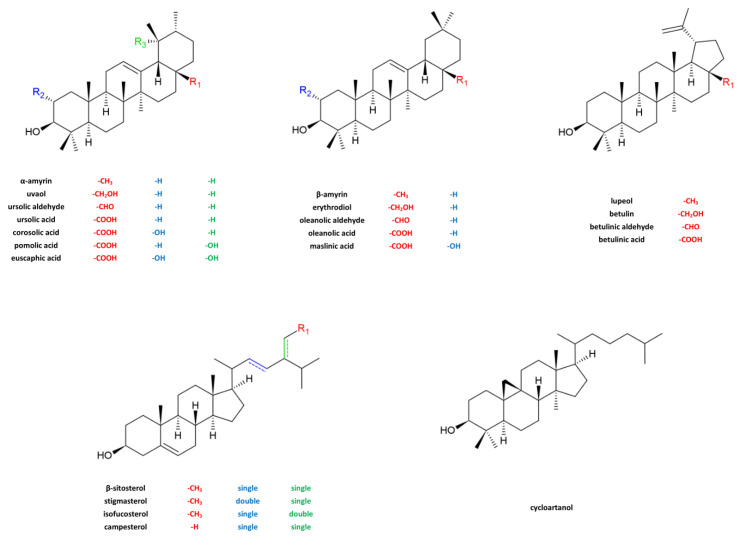
Triterpenoids quantified in apple fruits and leaves.

As stated below, the analyses of triterpenoids were conducted using GC-FID-MS. Three methods of analyte identification were applied simultaneously: use of analytical standards, examination of GC-MS spectra, and comparison of the results to samples of known composition from earlier studies. Meanwhile, a qualitative analysis was performed using an FID. Figure 1 presents the structures of the quantified compounds. Some of the compounds shown were not detected in the samples.

### 2.2. Triterpenoids in the Fruit

**Figure 2:**
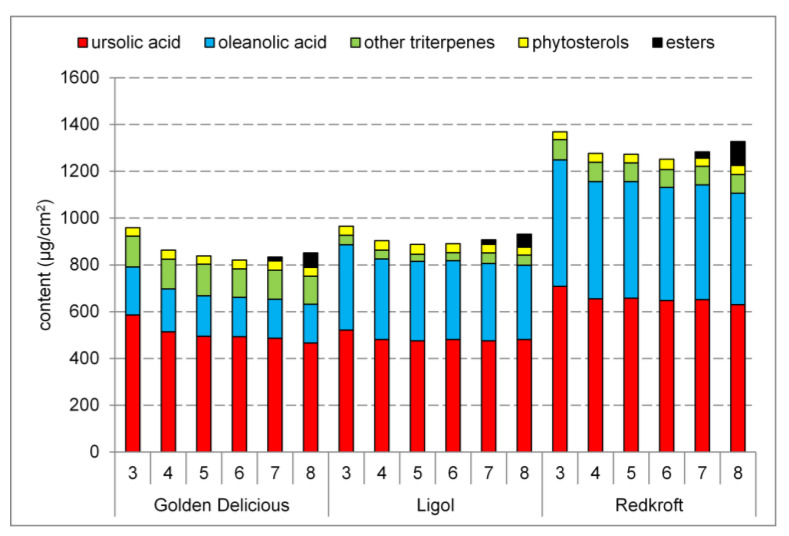
The changes in the triterpenoid content in fruits per unit area during the growth of three apple cultivars. Numbers on the horizontal axis denote the collection dates.

The cuticular waxes of all cultivars were dominated by ursolic acid and oleanolic acid. The content of minor triterpenic acids varied between the cultivars: ‘Redkroft’ contained only betulinic acid, ‘Ligol’ contained small amounts of pomolic acid, and ‘Golden Delicious’ contained betulinic acid as well as a wide spectrum of hydroxylated acids from the oleane and ursane families. Intermediates of triterpenic acid biosynthesis were detected at lower levels. The triterpenoid content was roughly constant, with slightly higher levels at the beginning of the growing season. Esterified forms of the triterpenes were detected at significant levels on the last two collection dates. Despite the lower content of free forms than ursolic acid, esters of oleanolic acid were more abundant in all cultivars. The phytosterol content was stable during the growing season and did not vary significantly between the cultivars. β-sitosterol was the most abundant compound from this group, with an approximately 90% share. Figure 2 presents the changes in the total triterpenoid content during the growing season, while Table 1 shows numerical data on the content of the most abundant constituents of the waxes in the last stage of development. Additionally, Table S1 in the Supporting Information presents all of the obtained data.

### 2.3. Triterpenoids in the Leaves

**Figure 3:**
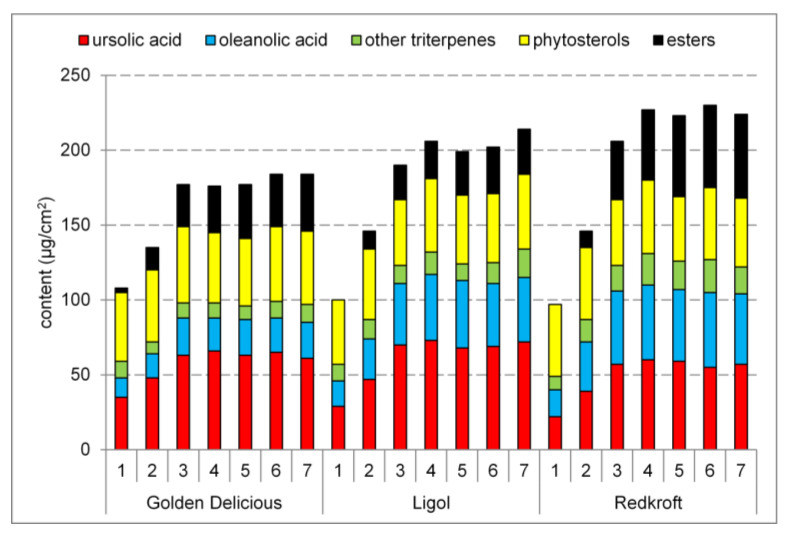
The changes in the triterpenoid content in leaves per unit area during the growth of three apple cultivars. Numbers on the horizontal axis denote the collection dates.

The cuticular waxes of the apple leaves contained the same analytes as those of the fruits; however, their levels were different. Ursolic acid and oleanolic acid were the most abundant triterpenic acids, although their content was almost an order of magnitude lower. The content of other triterpenic acids and neutral triterpenes decreased proportionally. The degree of esterification was much higher than in the fruits; additionally, esters were detected during the entire growing season. The phytosterol levels were stable and approximately 25% higher than in the fruits. The total triterpenoid content increased at the beginning of the season and was maintained in the later stages. Figure 3 presents the changes in the total triterpenoid content during the growing season, while Table 2 shows numerical data on the content of the most abundant constituents of the waxes in the last stage of development. Additionally, Table S2 in the Supporting Information presents all of the obtained data.

### 2.4. Antioxidative and Anti-Inflammatory Properties of Triterpenoids

**Figure 4:**
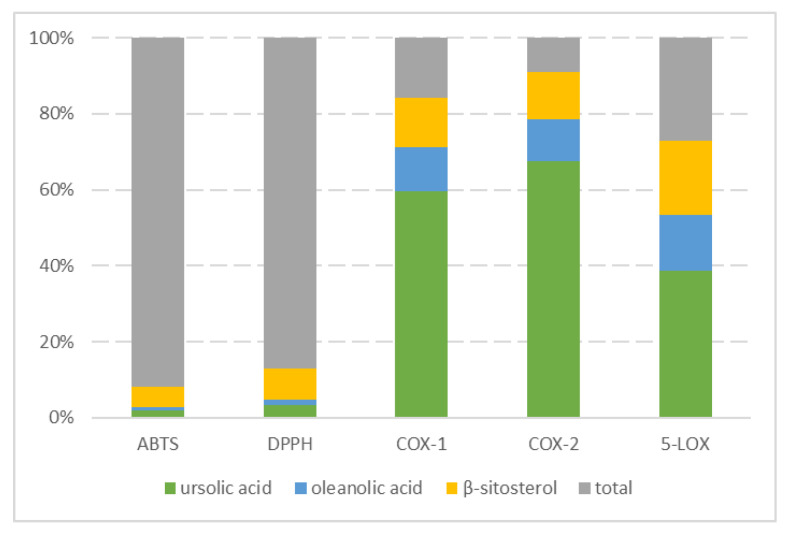
The contribution of the triterpenoids to the total antioxidative and anti-inflammatory activities of apple extracts. The activity of the apple extracts was compared with the theoretical activity of their selected constituents.

Three main triterpenoids of the apples as well as two phenolic compounds characteristic of these fruits were subjected to a series of analyses in order to evaluate their antioxidative and anti-inflammatory properties. Table 3 summarizes the IC_50_ values obtained for the pure compounds. Figure 4 compares the activities exhibited by apple extracts with the theoretical activity of their triterpenoid constituents.

### 2.5. Impact of Processing on Triterpenoid Content

The ‘Golden Delicious’ apples were subjected to lab-scale processing in order to evaluate the changes in the triterpenoid content during purée and juice production. Additionally, commercial samples of apple-based products were bought and analyzed. A summary of the results is presented in Table 4.

## 3. Discussion

### 3.1. Triterpenoids in the Fruit

The literature provides numerous publications dealing with the triterpenoid content in apples, including the analysis of the whole fruit, its selected parts, and processed apples; however, the results are often contradictory and difficult to compare due to the method of their presentation.

The typical triterpenic acid pattern was reported by Andre et al. in their study investigating 109 apple cultivars. They quantified the content of three triterpenic acids and found ursolic acid to be the most prevalent (median content in the peel of 1.32 mg g^−1^), followed by oleanolic acid (0.45 mg g^−1^) and betulinic acid (44 μg g^−1^). All cultivars except one showed a similar acid content profile: ursolic > oleanolic > betulinic, whilst the waxes of the ‘Merton Russet’ cultivar were dominated by betulinic acid [16]. Subsequent publications by these authors confirmed the findings, but also reported the presence of trans- and cis-caffeates of triterpenic acids in the apple cuticular waxes. These compounds were present in all the fruits tested; however, their content was significantly higher in russetted cultivars [21,25]. Similar results were obtained by other research teams. Butkevičiūte et al. analyzed the triterpenic acid content in six apple cultivars. In the peel, they found ursolic acid at levels of 4.03–6.43 mg g^−1^, oleanolic acid at 0.95–1.24 mg g^−1^, corosolic acid at 0.22–0.83 mg g^−1^, and betulinic acid at 39–83 μg g^−1^ (all expressed per dry weight), while the flesh contained only trace amounts of triterpenic acids [18]. Dashbaldan and colleagues reported the composition of cuticular waxes of the ‘Antonovka’ cultivar. The observed patterns were dominated by ursolic and oleanolic acid, while the minor triterpenic compounds included betulinic acid, corosolic acid, and oxo derivatives of ursolic and oleanolic acid [26]. Woźniak et al. analyzed the triterpene content in dried apple pomace obtained from an industrial plant and thus containing a mixture of cultivars. They found ursolic acid to be the most abundant (7.13 mg g^−1^), followed by oleanolic acid (1.59 mg g^−1^), pomolic acid (0.87 mg g^−1^), and neutral terpenoids at levels not exceeding 0.1 mg g^−1^ [27]. Considering the findings of Sut et al., the uniformity of the results can be connected with the common heritage of contemporary cultivars. The authors analyzed the phenolics and triterpenic acids in old Italian apple cultivars. Whereas commercial cultivars exhibited typical UA-dominated profiles with a total content in the dried peel of 10–50 mg g^−1^, the ancient varieties had a higher total content (25–70 mg g^−1^) and different qualitative and quantitative patterns: UA and OA were minor constituents, while large amounts of pomolic acid, maslinic acid, corosolic acid, and cuneataol were found [28]. The results obtained in our study are consistent with the data in the literature for commercial apple cultivars.

The aforementioned results refer to the mass of the peel, which, since it is mechanically removed, may differ between the studies and, therefore, is not the optimal method for expressing the triterpenoid content. Instead, we decided to express it per amount of area, in line with other authors. The series of papers by Lv and co-workers investigated the impact of cultivation and storage parameters on the content of terpenic compounds in apples. The first paper stated that cold storage does not affect triterpenoid levels [29], and the second reported that the cultivar and sun exposure can affect the triterpenoid content [30], while the last showed the impact of the root stock and harvest time [31]. The ursolic acid content varied slightly between the fruits; however, it was typically in the range of 250–600 μg cm^−2^, while oleanolic acid was at levels of 50–80 μg cm^−2^. Ju and Bramladge reported an increase in the content of all constituents of the cuticular waxes after ethylene treatment of ‘Delicious’ apples; the reported ursolic acid levels were in the range of 60–250 μg cm^−2^ [32]. The ursolic acid levels per area of fruit were also provided by Frighetto et al. in a paper focusing on the isolation of this compound. The authors analyzed four cultivars, obtaining a content of 210–820 μg cm^−2^ [33]. Significantly lower levels of ursolic and oleanolic acid in the ‘Florina’ and ‘Prima’ cultivars were reported by Leide et al., with 50 μg cm^−2^ and 5 μg cm^−2^, respectively [19]. The levels of ursolic acid (466–708 μg cm^−2^) and oleanolic acid (166–541 μg cm^−2^) presented in Table 1 are similar to those found in the aforementioned reports. The absence of literature on the levels of minor wax constituents makes a comparison impossible.

The literature includes studies implementing high-resolution mass spectrometry to identify the minor triterpenoid constituents of apple waxes. McGhie and colleagues detected 43 triterpenic acids and their derivatives in the peel of seven apple cultivars. In addition to monohydroxy acids (such as ursolic acid), di- and trihydroxy acids, oxo derivatives, and coumaric acid esters were also found; the results, however, were expressed only in relation to the total peak area [23]. Poirier et al. analyzed the content and partitioning of terpenoids in ‘Granny Smith’ apple cuticular waxes. In addition to the typical acidic and neutral triterpenes, the authors also found fatty acid esters of ursolic acid, uvaol, and α-amyrin. Remarkably, the first two were located mainly in the wax, while the latter was accumulated in the peel. The authors did not provide quantitative data on the content of the compounds in the samples [24]. Our study included an analysis of over 20 triterpenoids in free and esterified forms. An exact identification of the esters was not possible; however, due to the analytical approach, all bounded forms were quantified regardless of their chemical character. The presence of esterified forms of triterpenic acids and neutral triterpenes in apple waxes was confirmed.

It should be noted that contradictory results also appear in some papers. Bars-Cortina et al. analyzed phytochemicals in the flesh and peel of white- and red-fleshed apples. The peel contained ursolic (132–326 mg kg^−1^), hydroxyursolic (42–83 mg kg^−1^), euscaphic (9–127 mg kg^−1^), maslinic (13–30 mg kg^−1^), and betulinic (2–29 mg kg^−1^) acid; however, oleanolic acid was not reported in any of the nine cultivars [17]. Wildner and co-authors reported the ursolic and betulinic acid content in five apple cultivars from southern Brazil, but they did not present any data on oleanolic acid [20]. He and Liu investigated the constituents of the peel of ‘Red Delicious’ apples. They presented only low levels of neutral triterpenes and terpenic acid esters, while neither ursolic nor oleanolic acid was reported [22]. The discrepancies could be the result of focusing the analyses on particular compounds and therefore neglecting others. Misidentifications are possible as the compounds from the ursane and oleane groups create pairs of corresponding isomers.

The data in the literature on the sterol content in apples typically describe β-sitosterol-dominated patterns. The amount of this compound in fresh apples is in the range of 79–157 μg g^−1^ [34,35,36,37], while in pomace, it is 1147 μg g^−1^ [27]. Campesterol is typically the second most abundant sterol, although its levels are more than an order of magnitude lower [35,36]. The pattern of sterols in our samples was similar to the findings in the literature; however, the amounts expressed per weight of sample were much lower. Sterols are ubiquitous in cells, while our protocol only included an extraction of those present in the peel. Poirier and co-workers performed an in-depth analysis of the content and partitioning of steroids in ‘Granny Smith’ apple cuticular waxes. They found that, in addition to free sterols, acyl esters, glycosides, and acyl ester glycosides are also present. Most of these compounds were located in the peel rather than in the waxes. The authors did not provide quantitative data on the content of the compounds in the samples [24]. The results obtained in our study agree with the findings in the literature.

A few other teams reported changes in the triterpenoid content in other fruits. Dashbaldan and co-workers investigated the triterpenoid content in three phenological stages of the development of four edible berry species (*Vaccinium myrtillus*, *Vaccinium vitis-ideae*, *Arbutus unedo*, and *Lonicera caerulea*). The most abundant triterpenoids in their waxes were ursolic acid, oleanolic acid, α-amyrin, and β-sitosterol. Unfortunately, the results were expressed only in relation to the total mass of the extracted waxes. Nevertheless, the content of the majority of the analytes was constant during their development [38]. Salvador and colleagues focused their work on the phytochemicals of three elderberry (*Sambucus nigra* L.) cultivars. The cuticular waxes of all the cultivars were dominated by ursolic acid, which accounted for approx. 70% of the total content. The reported triterpenoid content decreased during the season; however, the results were only expressed as the dry mass of the fruit and, while the mass/area ratio changed during the growth of the fruit, are hard to compare with other findings [39].

### 3.2. Triterpenoids in the Leaves

According to our best knowledge, the literature contains only one report on the triterpenoid content in apple leaves. Bringe et al. analyzed adaxial (upper) surfaces and reported that the content of ursolic acid and oleanolic acid was in the range of 177–390 ng cm^−2^ and 20–97 ng cm^−2^, respectively [40]. The levels observed in our study were a few orders of magnitude higher. A more similar triterpenoid content was reported by Jetter et al., who analyzed the composition of cuticular waxes of *Prunus laurocerasus* leaves. The experiments showed a difference in the triterpenoid content between the abaxial (15 μg cm^−2^) and adaxial surfaces (5 μg cm^−2^). The publication reported the limited usability of mechanical methods of collecting surface wax for the analysis of triterpenoids, as they are present in the intracuticular part of the waxes [41].

Two publications by Pensec and colleagues investigated the cuticular waxes of grapes. The first one reported that oleanolic acid was the dominant compound in the fruit waxes of six cultivars. The changes in the composition of the waxes were also analyzed: a decrease in the triterpenoid content per gram of wax was reported; however, no recalculation considering fruit area was conducted [42]. The second publication focused on leaves and, surprisingly, reported a different pattern: the waxes were rich in lupeol and taraxeol, while oleanolic acid was one of the minor constituents [43]. In our study, the observed qualitative patterns were similar, but the levels of particular compounds differed between the fruits and the leaves.

### 3.3. Antioxidative and Anti-Inflammatory Properties of Triterpenoids

Three main triterpenes as well as two phenolic compounds typically found in apples were subjected to a series of assays to evaluate their potential health-promoting properties. The antioxidative capacity was evaluated via quantification of ABTS^•+^ and DPPH^•^ scavenging in simple chemical tests. Triterpenoids were inferior antioxidants compared to phenolic compounds; the assays showed that 4–7 times higher mass concentrations should be used to obtain a similar scavenging effect. The high antioxidative potential of phenolic compounds is a result of their structure; multiple double bonds as well as oxidation-prone hydroxyl and carbon groups allow them to accept several electrons per molecule [44,45]. The analyzed triterpenoids have a sole double bond; therefore, their potential in such tests is limited. Reports on antioxidative properties observed in vivo can be found in the literature [46,47,48,49], although the activity should be attributed to altered cell metabolism rather than a simple chemical reaction.

On the other hand, all three tests evaluating anti-inflammatory features showed a higher potential of triterpenes compared to phenolic compounds. The obtained IC_50_ values are similar to the literature findings [50,51,52], although their levels vary significantly between enzymes. Inhibition of cyclooxygenases can be considered promising, especially considering the COX-2/COX-1 ratio [53]. The high IC_50_ values for 5-LOX inhibition suggest that it could be impossible to obtain therapeutic concentrations in in vivo models without triggering cytotoxicity.

The contribution of triterpenoids to the overall antioxidative potential was minor. As stated before, phenolic compounds can be considered as the main antioxidants in apples, prevailing in both content and potential. Simultaneously, it was observed that triterpenic acids play a pivotal role in the inhibition of all of the tested pro-inflammatory enzymes, being responsible for up to 90% of the total activity. These findings should be considered as especially important since terpenes are often overlooked by food scientists and nutritionists investigating apples and apple-based products.

### 3.4. Impact of Processing on Triterpenoid Content

The levels of triterpenoids in the juices and purées were significantly lower than in the raw apples used for their production. In the case of ursolic and oleanolic acid, their content in the purée was approx. 3 times lower, while in the cloudy juice, their content was approx. 10 times lower. The content of β-sitosterol in the above products was 67% and 21% of the content in the apples, respectively. The contents of triterpenoids in the commercial products were similar to their laboratory equivalents; however, distinctions in the raw material and processing led to higher variance in the results for the commercial products. Meanwhile, in the clear juices, the levels of all analytes were below the limit of detection. These results are consistent with the literature; the presence of triterpenic acids is usually attributed to dried fruits and pomace, while their levels in juices are negligible [27,54]. The observed phenomena can be explained considering the low polarity of the triterpenoids. Their mass transfer from initial placement is insignificant; therefore, their content can be connected with the amount of apple tissue in products. Triterpenic acids are present mainly in the peel, the majority of which is discarded during processing; meanwhile, sterols are abundant in all cells, which results in higher retention in products.

The commercial products were subjected to thermal processing as part of their production. During the conventional pasteurization process, samples are kept at the temperature of 90–95 °C for 15–20 min, while newer applications are heading towards decreasing temperatures and process durations to limit the impact of heat on the sensory quality of the product and its bioactive compounds. For comparison, a significant rate of sitosterol oxidation is observed above 150 °C [55], while ursolic and oleanolic acids are even more stable in degradative processes occurring above 200 °C [56]. Therefore, it can be assumed that, in this case, temperature is not a significant factor.

The combination of the data levels of triterpenoids in various apple-based products and their anti-inflammatory properties can be used to draw another conclusion: the health benefits from the consumption of cloudy juices and purées will be superior to those from the consumption of clear juices. This supports earlier works highlighting the impact of fiber components in shaping the health-promoting activity of apple products [57,58].

## 4. Materials and Methods

### 4.1. Material

Three apple (*Malus domestica* Borkh.) cultivars varying in fruit peel coloration were selected for the experiment: ‘Golden Delicious’ (yellow), ‘Ligol’ (yellow with red blush), and ‘Redkroft’ (red). The plant material was collected in the experimental orchard of the National Institute of Horticulture Research in Dąbrowice (51°55′ N, 20°06′ E) during the 2017 growing season. The fruits and leaves were picked randomly from one tree per cultivar at regular intervals from April to September (eight times in total); Table 5 presents the dates when the material was collected. At least three fruits and six leaves were collected on each date to ensure that the samples were representative. The material collected was weighed, measured, and frozen at −18 °C prior to extraction. The Supporting Information presents the method of estimating the surface area [59].

The ‘Golden Delicious’ apples used during the processing and in vitro antioxidative and anti-inflammatory tests were bought in a supermarket in Warsaw, Poland. The apples were processed using lab-scale methods. A Miniprimer 9 blender (Braun, Kronberg im Taunus, Germany) was used for purée preparation, and a Robot Coupe J80 Ultra (Robot Coupe, Vincennes, France) was used for juice pressing, while the clear juice was obtained via centrifugation and subsequent filtration. Additionally, ten samples of each product, namely, clear juice, cloudy juice, and purée, were acquired from a local market in Warsaw.

### 4.2. Chemicals and Standards

The analytical standards of ursolic acid, oleanolic acid, betulinic acid, β-sitosterol, cholesteryl acetate, chlorogenic acid, and phloridzin were acquired from Merck (Darmstadt, Germany), while the standard of ursolic acid methyl ester was obtained from Carl Roth (Karlsruhe, Germany). Analytical-grade pyridine was acquired from Honeywell (Charlotte, NC, USA), while *N*, *O*-bis(trimethylsilyl)trifluoroacetamide with trimethylchlorosilane (BSTFA + 1% TMCS), *p*-anisaldehyde, and toluene were bought from Merck. Potassium hydroxide, acetic acid, sulfuric acid, and HPLC-grade methanol and chloroform were acquired from POCh (Gliwice, Poland). Hydrogen for chromatographic analysis was produced in situ by a Balston Hydrogen Generator (Parker Hannifin, Cleveland, OH, USA); other compressed gases were supplied by a local vendor.

### 4.3. Analysis of Triterpenoid Content

The multistep analytical procedure was conducted using an approach described by Pensec et al. [42]. The minor modifications that were implemented are described and justified in the descriptions of the individual stages.

The fruits and leaves were submerged in chloroform and stirred gently for 60 s. The amount of chloroform was selected to ensure at least 1 mL of solvent per square centimeter of fruit/leaf area. The extracts obtained were evaporated to dryness under reduced pressure using a Rotavapor R-300 vacuum dryer (Büchi, Flawil, Switzerland).

Part of the extracts obtained was subjected to alkaline hydrolysis using a protocol described by Woźniak et al. [60]. The reaction mixture was prepared by dissolving 0.75 g of potassium hydroxide in 1 mL of water, adding 4 mL of methanol, and dissolving the extract in 5 mL of toluene. The reaction was conducted for 60 min at 90 °C. The organic phase was collected, while the aqueous phase was re-extracted three times with toluene. The organic fractions were merged and evaporated to dryness. The original method included the separation of low polarity esters and their subsequent saponification and analysis [42]. The presence of hydrophobic fatty acid esters in the apple waxes is reported in the data in the literature [24]; however, reports describing the presence of esters of higher polarity, including esters of coumaric and caffeic acid, are also available [21,22,23]. Therefore, the selected approach allowed for the quantification of a wider array of terpenoid and triterpene derivatives.

The obtained extracts (both raw and hydrolyzed) were fractionated using preparative thin-layer chromatography (TLC). The samples were applied to TLC silica gel 60 glass plates (10 cm × 20 cm) (Merck, Darmstadt, Germany) and developed in a chloroform/methanol (97:3, *v*/*v*) mixture. The analytical standards of oleanolic acid and β-sitosterol were used to localize fractions containing triterpenic acids (R_F_ 0.2–0.3) and steroids and neutral triterpenes (RF 0.3–0.9), respectively. Due to safety concerns, the standards were visualized by spraying them with an anisaldehyde sulfuric acid reagent (recipe reported in the Supporting Information) and heating [61], instead of the use of 50% sulfuric acid described in the original method. Zones of the plate coating containing the desired fractions were scraped, and, subsequently, the analytes were rinsed with chloroform/methanol (2:1, *v*/*v*).

The fractions containing triterpenic acids were subjected to derivatization prior to the chromatographic analysis. The silylation protocol presented by Sánchez Ávila et al. [62] was used, instead of the original derivatization method utilizing diazomethane, which can be hazardous. An amount of 1 mL of the sample was placed in a 1.5 mL reaction tube and evaporated under a gentle stream of nitrogen. Afterwards, 600 μL of pyridine and 300 μL of BSTFA + 1% TMCS were added, and the tube was heated at 80 °C for 2 h.

Quantitative analyses of the samples were conducted using a Varian 430-GC gas chromatograph with a built-in flame ionization detector (FID) and CP-8400 autosampler (Varian, Palo Alto, CA, USA). The separations were conducted using a DB-5, 30 m × 0.25 mm, 0.25 μm column (Agilent Technologies, Palo Alto, CA, USA) that was eluted with helium at a flowrate of 1.0 mL min^−1^. Samples (2.5 μL) were injected using a 1:10 split ratio and an injector temperature of 280 °C. Silylated triterpenic acids were analyzed under isothermal conditions at 280 °C for 60 min, while steroids and neutral triterpenes were analyzed using a temperature program: initial temperature of 160 °C for 2 min, which was increased at 5 °C min^−1^ to a final temperature of 280 °C that was held for a further 44 min. The detector was kept at a temperature of 300 °C, while the gas flows were 25 mL min^−1^ of N_2_, 30 mL min^−1^ of H_2_, and 300 mL min^−1^ of air. An internal standard (cholesteryl acetate) was added to each sample prior to the analysis.

The identification of the analytes was performed based on a comparison of their retention times with the standards. The five-point calibration curves showed good linearity in the tested range; the LOQ value for all analytes was set to 0.2 μg cm^−2^. Additionally, selected samples were subjected to GC-MS analysis, and the obtained mass spectra were compared with the data in the literature. The GC-MS analyses were conducted using an Agilent 7890A gas chromatograph and 5975C mass spectrometric detector (both Agilent Technologies). The separation parameters were the same as those described above for the GC-FID analysis; the mass spectrometer worked with an energy of ionization of 70 eV and an *m/z* range of 33–500.

### 4.4. Antioxidative and Anti-Inflammatory Properties

The selected peel extracts as well as the standards of triterpenic compounds were analyzed for their antioxidative and anti-inflammatory properties. Due to their hydrophobic character, the samples were dissolved in dimethyl sulfoxide (DMSO). The content of DMSO in the reaction media in all the tests described below was 1% for all the tested samples as well as the controls.

The scavenging activity against ABTS^•+^ was measured with a protocol described by Re et al. [63]. A solution of 7 mM of ABTS and 2.45 mM of potassium persulfate was used for the generation of radical cations. After overnight incubation in darkness, the solution was diluted with ethanol to obtain an absorbance of approx. 0.7 at 734 nm. Then, 25 µL samples were mixed with 2.5 mL of ABTS^•+^ solution and incubated for 6 min. The scavenging of ABTS^•+^ molecules was measured spectrophotometrically at 734 nm. Trolox was chosen as a reference antioxidant.

A method described by Yen and Chen was used to determine the activity against DPPH^•^ [64]. Briefly, 0.1 mL samples were mixed with 2 mL of DPPH^•^ solution (1 mM) and incubated at room temperature for 30 min. The scavenging of DPPH^•^ radicals was quantified spectrophotometrically at 517 nm. Trolox was chosen as a reference antioxidant.

The anti-inflammatory activity was evaluated using commercial kits for three enzymes of biological activity: cyclooxygenases 1 and 2 (COX-1 and COX-2) and 5-lipoxygenase (5-LOX), according to the instructions of the producers. Inhibition of the cyclooxygenases was measured using kits from Cayman Chemicals (Ann Arbor, MI, USA; items 701070 and 701080, respectively), while 5-LOX inhibition was measured using a kit from abcam (Cambridge, UK; item no. ab284521).

### 4.5. Statistics

Three independent samples were analyzed for each of the matrices. The data were analyzed using Statistica 7.1 software (StatSoft, Tulsa, OK, USA). ANOVA with a post hoc Tukey test at α = 0.05 was used to determine the statistical significance of the differences.

## Figures and Tables

**Table 1:** The content of selected triterpenoids in apple fruits in the last stage of development. Data in Roman script represent unbounded analytes, while data in italic script correspond to their esterified forms. Letters represent the statistical significance of the differences between cultivars (Tukey test, α = 0.05).

Content (μg cm^−2^)	‘Golden Delicious’	‘Ligol’	‘Redkroft’
Ursolic acid	466.2 ± 24.0 c	581.0 ± 14.2 b	630.4 ± 11.0 a
	*13.1 ± 6.4 b*	*12.1 ± 2.8 b*	*31.5 ± 3.1 a*
Oleanolic acid	166.1 ± 13.2 c	317.8 ± 19.8 b	476.1 ± 7.7 a
	*39.4 ± 2.0 b*	*38.1 ± 5.4 b*	*63.1 ± 3.4 a*
Betulinic acid	30.2 ± 4.1 b	nd	52.1 ± 7.1 a
	*1.4 ± 0.7 a*	*nd*	*4.3 ± 2.0 a*
Pomolic acid	49.4 ± 5.7 a	10.2 ± 2.5 b	nd
	*4.1 ± 2.4*	*nd*	*nd*
Corosolic acid	10.2 ± 1.5	nd	nd
	*1.1 ± 0.4*	*nd*	*nd*
α-amyrin	9.0 ± 0.7 a	8.2 ± 0.5 ab	6.3 ± 0.5 b
	*nd*	*nd*	*nd*
Uvaol	4.1 ± 0.7 a	5.1 ± 0.8 a	5.3 ± 0.6 a
	*nd*	*nd*	*nd*
β-amyrin	7.1 ± 0.9 a	5.0 ± 1.1 ab	3.3 ± 0.2 b
	*nd*	*nd*	*nd*
Erythrodiol	3.2 ± 0.5 a	2.1 ± 0.3 a	2.9 ± 0.2 a
	*nd*	*nd*	*nd*
β-sitosterol	35.2 ± 1.6 a	33.3 ± 1.0 a	37.4 ± 1.9 a
	*1.4 ± 0.3 a*	*1.7 ± 0.4 a*	*1.4 ± 0.4 a*
Campesterol	1.4 ± 0.2 a	1.0 ± 0.1 a	1.2 ± 0.2 a
	*nd*	*nd*	*nd*

nd—not detected (≤0.2 μg cm^−2^).

**Table 2:** The content of selected triterpenoids in apple leaves in the last stage of development. Data in Roman script represent unbounded analytes, while data in italic script correspond to their esterified forms. Letters represent the statistical significance of the differences between cultivars (Tukey test, α = 0.05).

Content (μg cm^−2^)	‘Golden Delicious’	‘Ligol’	‘Redkroft’
Ursolic acid	61.2 ± 4.1 a	71.9 ± 5.0 a	57.3 ± 3.2 a
	*12.1 ± 1.4 b*	*13.9 ± 1.2 ab*	*18.3 ± 1.9 a*
Oleanolic acid	24.0 ± 2.0 b	43.4 ± 1.6 a	46.5 ± 2.4 a
	*18.4 ± 1.2 b*	*12.1 ± 0.9 c*	*31.5 ± 0.7 a*
Betulinic acid	1.6 ± 0.2 b	nd	8.2 ± 1.4 a
	*nd*	*nd*	*0.9 ± 0.4*
Pomolic acid	1.4 ± 0.2 a	0.7 ± 0.0 b	nd
	*0.5 ± 0.2*	*nd*	*nd*
Corosolic acid	nd	nd	nd
	*nd*	*nd*	*nd*
α-amyrin	4.1 ± 0.6 ab	5.2 ± 0.3 a	3.7 ± 0.5 b
	*0.6 ± 0.2 a*	*0.8 ± 0.0 a*	*0.5 ± 0.2 a*
Uvaol	2.1 ± 0.4 a	1.7 ± 0.3 a	1.9 ± 0.3 a
	*nd*	*nd*	*nd*
β-amyrin	2.0 ± 0.2 b	4.9 ± 0.7 a	2.6 ± 0.4 b
	*nd*	*0.6 ± 0.1*	*nd*
Erythrodiol	1.1 ± 0.3 ab	1.4 ± 0.2 a	0.8 ± 0.0 b
	*nd*	*nd*	*nd*
β-sitosterol	45.8 ± 1.6 a	48.3 ± 2.1 a	44.7 ± 1.3 a
	*2.6 ± 0.4 a*	*3.4 ± 0.5 a*	*2.5 ± 0.2 a*
Campesterol	1.7 ± 0.4 a	1.2 ± 0.2 a	1.0 ± 0.3 a
	*nd*	*nd*	*nd*

nd—not detected (≤0.2 μg cm^−2^).

**Table 3:** Antioxidative and anti-inflammatory properties of the main triterpenoids and phenolic compounds found in the apples. The results are expressed per dry mass. All results are presented as IC_50_ expressed in mg L^−1^.

Test	Ursolic Acid	Oleanolic Acid	β-Sitosterol	Chlorogenic Acid	Phloridzin	Apple Extract
ABTS^•+^	163 ± 8	155 ± 7	130 ± 4	23 ± 2	34 ± 4	140 ± 8
DPPH^•^	94 ± 3	96 ± 5	88 ± 5	21 ± 3	23 ± 3	82 ± 9
COX-1	52 ± 4	104 ± 6	542 ± 6	1047 ± 73	960 ± 61	205 ± 12
COX-2	31 ± 3	73 ± 7	382 ± 4	612 ± 31	644 ± 38	144 ± 10
5-LOX	717 ± 43	641 ± 22	1740 ± 52	>5000	>5000	2084 ± 301

**Table 4:** Content of the main triterpenoids in apple products from laboratory processing (triplicate production process) and commercial sale (ten distinct products per category). The results are expressed per wet mass. Letters represent the statistical significance of the differences between groups (Tukey test, α = 0.05).

Content (mg L^−1^) or (mg kg^−1^)	Ursolic Acid	Oleanolic Acid	β-Sitosterol
Apple	56.1 ± 3.1 a	24.1 ± 1.3 a	80.5 ± 3.1 a
Purée (laboratory)	19.6 ± 2.1 b	8.0 ± 1.1 c	53.6 ± 4.2 b
Cloudy juice (laboratory)	6.2 ± 0.9 c	2.8 ± 0.4 d	17.0 ± 2.9 d
Clear juice (laboratory)	<0.1 d	<0.1 e	<0.1 e
Purée (commercial)	22.3 ± 3.9 b	15.2 ± 4.0 b	34.1 ± 11.1 c
Cloudy juice (commercial)	4.1 ± 2.0 c	2.3 ± 0.5 d	14.1 ± 5. d
Clear juice (commercial)	<0.1 d	<0.1 e	<0.1 e

**Table 5:** The dates of sample collection. The pluses and minuses denote whether the material was available on that day.

Term	1	2	3	4	5	6	7	8
Date	21 April	12 May	2 June	27 June	19 July	14 August	8 September	29 September
Fruits	-	-	+	+	+	+	+	+
Leaves	+	+	+	+	+	+	+	-

## Data Availability

Data is contained within the article or supplementary material.

## Supplementary Materials

The following Supporting Information can be downloaded at: https://www.mdpi.com/article/10.3390/molecules28062584/s1. Table S1. Content of triterpenoids in apple fruit. Only compounds detected in at least one sample were listed. All data in μg/cm^2^. nd—not detected (≤0.2 μg/cm^2^) Table S2. Content of triterpenoids in apple leaves. Only compounds detected in at least one sample were listed. All data in μg/cm2. nd—not detected (≤0.2 μg/cm^2^).
