# Supplementary material for: Triterpenoids of Three Apple Cultivars—Biosynthesis, Antioxidative and Anti-Inflammatory Properties, and Fate during Processing
Source: Molecules. 2023 Mar 13;28(6):2584. doi: 10.3390/molecules28062584 (PMC10058748; doi:10.3390/molecules28062584)
Supplement: Supplementary file 1 [file molecules-28-02584-s001.zip › supplementary material.pdf]

Evaluation of leaf area

Two main dimensions were measured for each leaf – length (l) and width (w). The surfaces of leaves were assumed to be matching ellipses, while sides were ignored due to neglectable thickness of the leaves. The surface are of each of the fruit was estimated as:

$$A = \frac{\pi \cdot l \cdot w}{2}$$

Evaluation of fruit area

Two main dimensions were measured for each fruit – height (h) and diameter (d). The fruit were assumed to be spheroids and, according to their height - diameter ratio, were classified to three groups: spherical (h = d), prolate (h > d), and oblate (h < d). The surface area of each of the fruit was estimated using one of the following equations:

- spherical fruit:

$$A = \pi \cdot d^2$$

- prolate fruit:

$$A = \frac{\pi \cdot d}{2} \cdot \left(1 + \frac{1-e^2}{e} \cdot artanh\,(e)\right),\ \text{where}\ \ e^2 = 1 - d^2/h^2$$

- oblate fruit:

$$A = \frac{\pi \cdot d}{2} \cdot \left(1 + \frac{c}{a \cdot e} \cdot arcsin\,(e)\right),\ \text{where}\ \ e^2 = 1 - h^2/d^2$$

Table S1. Content of triterpenoids in apple fruit. Only compounds detected in at least one sample were listed. All data in μg/cm^2. nd - not detected (≤ 0.2 μg/cm^2)

Golden Delicious							
compound		date					
		3	4	5	6	7	8
ursane family							
α-amyrin	free	8.1	7.4	7.9	6.3	8.5	9.0
uvaol	free	6.1	5.3	6.0	7.1	5.4	4.1
ursolic aldehyde	free	0.9	1.2	0.9	1.1	1.0	1.3
ursolic acid	free	585.7	514.2	494.5	493.0	486.1	446.2
	bonded	nd	nd	nd	nd	5.3	13.1
corosolic acid	free	8.1	7.6	6.9	7.7	7.1	10.2
	bonded	nd	nd	nd	nd	nd	1.1
pomolic acid	free	61.4	53.5	48.1	52.0	51.4	49.4
	bonded	nd	nd	nd	nd	1.1	4.1
euscaphic acid	free	4.2	2.9	4.0	3.6	3.1	3.7
oleane family							
β-amyrin	free	8.4	6.9	7.0	7.7	6.6	7.1
erythrodiol	free	5.2	4.8	3.9	3.5	3.3	3.2
oleanolic aldehyde	free	1.3	0.8	0.7	1.1	1.5	1.2
oleanolic acid	free	205.2	183.3	172.9	168.4	166.8	166.1
	bonded	nd	nd	nd	nd	8.2	39.4
maslinic acid	free	2.1	3.3	2.8	2.3	3.1	2.8
lupane family							
lupeol	free	1.4	0.8	0.9	1.2	1.4	1.4
betulinic acid	free	37.2	33.1	28.9	30.6	26.1	30.2
	bonded	nd	nd	nd	nd	0.4	1.4
sterols							
β-sitosterol	free	34.1	36.4	33.7	37.0	38.8	35.2
	bonded	nd	nd	nd	nd	0.8	1.4
stigmasterol	free	nd	nd	0.4	0.5	nd	0.4
campesterol	free	1.5	1.1	1.4	1.2	1.3	1.4
cycloartanol	free	0.4	0.6	0.4	0.5	0.7	0.6

Table S1. Continuation

Ligol							
compound		date					
		3	4	5	6	7	8
ursane family							
α-amyrin	free	10.4	9.7	7.9	8.3	8.6	8.2
uvaol	free	6.8	5.3	6.1	4.8	4.2	5.1
ursolic aldehyde	free	2.0	1.3	1.4	1.7	1.7	1.4
ursolic acid	free	621.0	581.4	575.3	580.8	574.6	581.0
	bonded	nd	nd	nd	nd	5.1	12.1
pomolic acid	free	16.1	14.3	12.2	11.1	11.0	10.2
oleane family							
β-amyrin	free	6.1	4.3	5.2	6.0	6.4	5.0
erythrodiol	free	2.8	1.7	2.1	2.3	2.0	2.1
oleanolic aldehyde	free	1.9	2.0	1.3	1.2	1.4	1.3
oleanolic acid	free	364.9	344.4	340.0	336.9	330.8	317.8
	bonded	nd	nd	nd	nd	10.5	38.1
sterols							
β-sitosterol	free	36.4	39.9	40.2	37.4	35.1	33.3
	bonded	nd	nd	nd	nd	0.5	1.7
stigmasterol	free	0.9	1.1	1.3	1.0	0.9	0.7
campesterol	free	1.2	0.9	0.9	1.0	1.3	1.0

Table S1. Continuation

Redkroft							
compound		date					
		3	4	5	6	7	8
ursane family							
α-amyrin	free	7.1	6.0	5.8	7.3	6.2	6.3
uvaol	free	6.4	5.7	7.1	6.2	5.8	5.3
ursolic aldehyde	free	2.2	3.1	3.4	3.0	2.7	3.1
ursolic acid	free	708.1	655.2	658.3	647.8	651.5	630.4
	bonded	nd	nd	nd	nd	7.0	31.5
oleane family							
β-amyrin	free	4.2	4.0	3.8	3.1	3.5	3.3
erythrodiol	free	2.2	2.6	2.4	2.6	2.9	2.9
oleanolic aldehyde	free	1.3	1.0	1.3	1.6	1.6	1.4
oleanolic acid	free	541.1	501.4	497.8	483.0	491.0	476.1
	bonded	nd	nd	nd	nd	18.4	63.1
lupane family							
lupeol	free	2.9	1.8	1.8	2.2	1.9	2.2
betulin	free	2.0	1.3	1.6	1.5	1.9	1.7
betulinic acid	free	64.3	59.1	53.4	55.0	48.6	52.1
	bonded	nd	nd	nd	nd	0.7	4.3
sterols							
β-sitosterol	free	35.1	36.2	34.8	41.3	34.2	37.4
	bonded	nd	nd	nd	nd	0.4	1.4
campesterol	free	1.4	1.1	1.5	1.3	1.2	1.2
cycloartanol	free	1.0	0.8	0.8	1.2	1.0	0.9

Table S2. Content of triterpenoids in apple leaves. Only compounds detected in at least one sample were listed. All data in μg/cm^2. nd - not detected (≤ 0.2 μg/cm^2)

Golden Delicious								
compound		date						
		1	2	3	4	5	6	7
ursane family								
α-amyrin	free	3.6	4.2	4.5	3.8	4.0	3.8	4.1
	bonded	nd	0.4	0.6	0.7	0.4	0.4	0.6
uvaol	free	2.0	1.8	2.4	2.4	2.2	2.5	2.1
	bonded	nd	nd	nd	0.2	nd	0.3	nd
ursolic acid	free	35.4	48.1	62.6	66.1	63.3	65.0	61.2
	bonded	nd	3.1	10.6	11.7	12.5	11.9	12.2
pomolic acid	free	nd	0.8	1.1	1.6	1.5	1.6	1.4
	bonded	nd	nd	nd	nd	0.2	0.3	nd
oleane family								
β-amyrin	free	1.8	2.4	1.9	1.7	2.1	1.9	2.0
	bonded	nd	nd	nd	nd	0.4	0.4	nd
erythrodiol	free	0.8	1.0	1.1	0.8	1.2	1.1	1.1
oleanolic acid	free	13.4	16.5	25.1	22.5	23.9	23.1	24.0
	bonded	nd	4.7	16.2	17.1	19.0	18.4	18.4
lupane family								
betulinic acid	free	0.5	1.0	1.5	1.4	1.5	1.7	1.6
sterols								
β-sitosterol	free	44.3	46.1	48.3	44.0	43.2	47.5	45.8
	bonded	nd	1.0	1.7	2.5	2.8	2.1	2.6
stigmasterol	free	1.0	0.6	0.8	1.0	1.1	0.8	0.7
campesterol	free	1.5	1.3	1.7	1.8	1.6	1.5	1.7
cycloartanol	free	0.6	0.4	0.6	0.3	0.5	0.4	0.5

Table S2. Continuation

Ligol								
compound		date						
		1	2	3	4	5	6	7
ursane family								
α-amyrin	free	6.3	5.1	5.8	7.0	6.1	5.6	5.2
	bonded	nd	nd	0.4	0.7	1.1	1.0	0.8
uvaol	free	1.6	2.1	2.3	2.0	1.8	2.1	1.7
	bonded	nd	nd	nd	nd	0.3	0.3	nd
ursolic aldehyde	free	nd	nd	0.4	nd	nd	0.3	nd
ursolic acid	free	29.2	46.8	69.9	73.1	68.5	69.0	71.9
	bonded	nd	4.2	11.1	13.2	14.0	12.8	13.9
pomolic acid	free	nd	nd	0.4	0.5	nd	0.6	0.7
oleane family								
β-amyrin	free	4.0	5.1	5.2	5.0	4.8	4.6	4.9
	bonded	nd	nd	0.5	0.4	nd	0.4	0.6
erythrodiol	free	0.9	1.3	1.2	1.6	1.3	1.5	1.4
oleanolic acid	free	17.1	26.8	41.0	44.3	45.5	41.9	43.4
	bonded	nd	3.2	10.0	12.3	12.5	11.8	12.1
sterols								
β-sitosterol	free	42.7	44.8	42.1	46.3	43.0	44.1	48.3
	bonded	nd	0.6	1.3	2.6	2.8	3.2	3.4
stigmasterol	free	0.6	0.9	1.1	0.8	1.0	0.9	0.7
campesterol	free	1.3	1.1	1.1	1.0	1.2	1.0	1.2

Table S2. Continuation

Redkroft								
compound		date						
		1	2	3	4	5	6	7
ursane family								
α-amyrin	free	3.5	4.0	3.5	3.1	3.9	4.2	3.7
	bonded	nd	0.4	0.6	0.4	nd	0.5	0.5
uvaol	free	1.7	1.1	1.0	1.7	1.5	1.6	1.9
	bonded	nd	nd	0.5	nd	0.4	nd	nd
ursolic acid	free	22.4	38.6	57.2	60.3	58.7	55.5	57.3
	bonded	nd	9.5	16.8	15.7	19.0	17.6	18.3
oleane family								
β-amyrin	free	2.3	3.2	2.1	1.8	2.2	2.1	2.6
	bonded	nd	nd	nd	nd	nd	0.4	nd
erythrodiol	free	0.6	nd	0.5	0.7	0.7	1.0	0.8
oleanolic acid	free	18.2	33.3	49.1	50.0	48.4	49.7	46.5
	bonded	nd	14.5	29.6	33.0	28.5	28.9	31.5
lupane family								
lupeol	free	nd	nd	0.5	0.5	0.4	0.4	nd
betulinic acid	free	6.0	7.6	8.2	8.5	8.1	9.0	8.2
	bonded	nd	0.6	0.6	0.4	0.5	0.5	0.9
sterols								
β-sitosterol	free	46.2	44.1	42.5	46.0	42.5	46.0	44.7
	bonded	nd	1.2	1.9	2.3	2.7	2.3	2.5
campesterol	free	0.8	1.1	1.2	1.3	1.1	1.1	1.0
cycloartanol	free	0.4	0.3	nd	0.5	0.4	0.4	0.5
